# Antioxidant and lipid-lowering effects of freeze-dried kimchi cabbage and onion mediated via inhibition of adipogenesis in 3T3-L1 adipocytes

**DOI:** 10.29219/fnr.v69.11101

**Published:** 2025-10-13

**Authors:** Ye-Rang Yun, Wooje Lee, Sung Wook Hong

**Affiliations:** World Institute of Kimchi, Nam-Gu, Gwangju, Republic of Korea

**Keywords:** antioxidant effect, freeze-drying, kimchi cabbage, lipid accumulation, obesity-associated mRNA/protein expression

## Abstract

**Background:**

Kimchi exhibits various beneficial effects on human health, which are ascribed to its ingredients, bioactive compounds, lactic acid bacteria, and metabolites.

**Objective:**

To explore the antioxidant and lipid-lowering effects of individual ingredients of freeze-dried kimchi in 3T3-L1 adipocytes.

**Design:**

The lipid-lowering effects of six kimchi ingredients were investigated using 3T3-L1 adipocytes. Their antioxidant activities, cytotoxicity, and the effect on triglyceride (TG) content, lipid accumulation, and obesity-associated biomarker expressions were examined.

**Results:**

Freeze-dried ginger exhibited the highest antioxidant activity, followed by kimchi cabbage and onion. Freeze-dried garlic and green onion showed cytotoxicity, and the TG content in freeze-dried ginger-treated cells was similar to that of the control. Freeze-dried kimchi cabbage- and onion-treated cells exhibited increased antioxidant activities, low cell toxicity, and remarkable effects on the TG content. Selected freeze-dried kimchi cabbage and onion significantly inhibited lipid accumulation and decreased the expression of obesity-associated messenger RNA (mRNA) and proteins, with freeze-dried kimchi cabbage being more efficient (*P* < 0.05).

**Discussion:**

The six kimchi ingredients showed differences in anti-obesity effects, and these effects may be related to antioxidant properties.

**Conclusion:**

Freeze-dried kimchi cabbage exhibited the most pronounced antioxidant and lipid-lowering effects among the six kimchi ingredients tested, highlighting the potential applications of kimchi in obesity-associated metabolic pathway research.

**Graphical Abstract:**

**Figure:**
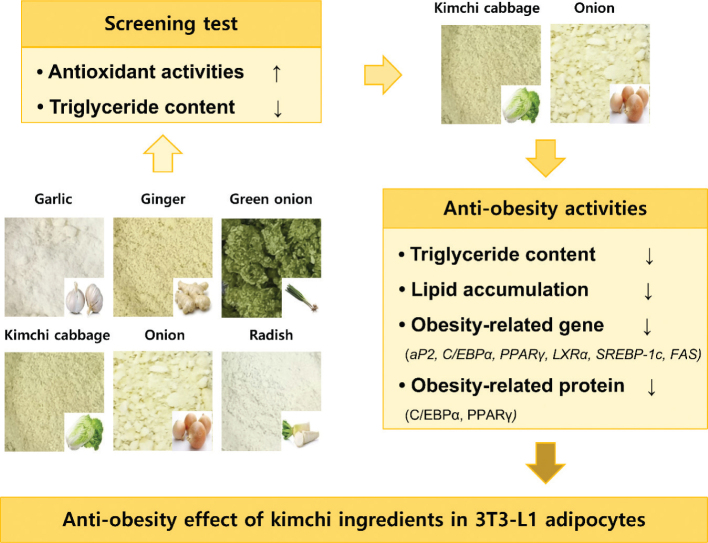

## Popular scientific summary

- Freeze-dried kimchi cabbage and onion exhibited antioxidant and lipid-lowering effects.
- These ingredients can control obesity by inhibiting lipid accumulation and modulating obesity-associated mRNA/protein expression.
- Our results indicate that the health benefits of kimchi are primarily attributed to its actual ingredients.

The health functionalities of kimchi have been consistently demonstrated (1–6). These effects have been ascribed to the ingredients, bioactive compounds, lactic acid bacteria (LAB), metabolites, and kimchi itself. In a previous study, the bioactive kimchi compounds inhibited tunicamycin-induced hepatic steatosis, suggesting its potential use in preventing non-alcoholic fatty liver disease (5). Similar effects were observed with freeze-dried kimchi treatment (6). Kimchi LAB remarkably modulates obesity *in vitro* (1). In addition, kimchi combined with red yeast rice has demonstrated an anti-obesity effect by modulating biomarker expression in the liver X receptor α (LXRα) and peroxisome proliferator-activated receptor γ (PPARγ) pathways (2). Similarly, functional LAB-fermented kimchi modulates gut microbiome composition, which is a possible mechanism of action underlying its anti-obesity effect (4). While previous studies have investigated the functionalities of kimchi, its bioactive compounds, and LAB, research focusing on individual ingredients of kimchi, except for red pepper (7, 8), is limited. Hence, further studies on kimchi ingredients are warranted.

Kimchi contains various ingredients, including kimchi cabbage, radish, and red pepper. The vegetables used in kimchi not only contain bioactive compounds but also exhibit functional properties, thereby enhancing the overall functionality of kimchi (9, 10). To date, only extracts or solvent fractions of these ingredients have been used to demonstrate the health benefits of kimchi ingredients. Representatively, capsaicin in peppers (*Capsicum annuum*) exerts antioxidant and anti-obesity effects (11), and studies examining the seeds, leaves, and capsaicin of peppers have been conducted (7, 8). Kim et al. reported that a water extract of red pepper seeds controlled obesity by suppressing obesity-associated biomarkers in obese mice (7). Water-soluble red pepper leaf extract also suppresses obesity by inhibiting fat accumulation and mRNA expression (8). Another kimchi ingredient, kimchi cabbage (Chinese cabbage), shows anti-bacterial and anti-cancer effects attributed to sulforaphane (12). Ge et al. revealed that sulforaphane exhibits an anti-cancer effect by inhibiting the proliferation of gastric cancer stem cells (13).

Freeze-drying is a non-thermal process that retains most of the bioactive compounds, enzymes, and antioxidants present in the fresh material. Therefore, although the physical form is altered, the chemical and nutritional integrity of the ingredients remains largely intact. This method also allows for standardized dosing, reproducibility, and reduced variability—key advantages for *in vitro* studies (14, 15). Although freeze-dried ingredients are not in their raw physical state, this processing method preserves the full spectrum of bioactive compounds, allowing them to be used as a practical and scientifically valid proxy for whole-form evaluation *in vitro* settings.

Previously, the anti-obesity effect of freeze-dried ripe kimchi has been elucidated in cell and animal models. We hypothesized that the functionality of kimchi can be attributed to kimchi ingredients. Hence, we aimed to explore the functionality of whole kimchi ingredients, rather than that of individual extracts or fractions. The objective was to compare the effect of freeze-dried kimchi ingredients on antioxidant activity, cell viability, and triglyceride (TG) content in 3T3-L1 adipocytes to determine the effects of selected kimchi ingredients that were explored on lipid accumulation and obesity-associated biomarker expression.

## Materials and methods

### Reagents

Antioxidant activity standards (gallic acid and quercetin), total antioxidant capacity (TAC) kit, and adipocyte differentiation markers (3-isobutyl-1-methylxanthine [IBMX], dexamethasone, insulin, and oil red O [ORO] solution) were acquired from Sigma Aldrich, St. Louis, MO, USA. Cell culture reagents were acquired from Gibco, Gaithersburg, MD, USA. Ferric-reducing antioxidant power (FRAP) kits (Ann Arbor, MI, USA), cell counting kit-8 (CCK-8, Dojindo, Japan), TG assay kits (Asan Pharmaceutical, Seoul, South Korea), and TRIzol^TM^ reagent (Invitrogen, CA, USA) were obtained. TOPScript^TM^ cDNA Synthesis Kit and SYBR Green premix were acquired from Enzynomics Inc., Daejeon, South Korea. Radioimmunoprecipitation assay (RIPA) buffer (Thermo Fisher Scientific, Waltham, MA, USA), antibodies (Cell Signaling Technology, Danvers, MA, USA), and an enhanced chemiluminescence (ECL) system (ECL Advance, GE Healthcare; Hatfield, UK) were also used.

### Preparation of kimchi ingredients

Kimchi ingredients – garlic, ginger, green onion, kimchi cabbage, onion, and radish – were acquired from an agriculture and marine market (Gwangju, South Korea). All kimchi ingredients were washed, cut, and subjected to hot-air drying at 50°C using a drying oven (VS-1203P3, Vision Scientific Co., Daejeon, South Korea). Subsequently, they were freeze-dried at -70°C using a freeze dryer (Operon FDT-8632, Seoul, South Korea). After drying each kimchi ingredient (1 kg), the ingredients were ground and weighed. For cellular experiments, freeze-dried kimchi ingredients were dissolved in dimethyl sulfoxide (DMSO) and filtered using a 0.45 μm syringe filter (Sartorius, Germany). A 100 mg/mL stock solution was prepared and diluted with Dulbecco’s modified Eagle’s medium (DMEM) for use.

### Antioxidant activity analysis

The antioxidant activities of hot-air-dried and freeze-dried kimchi ingredients were determined by measuring total phenol content (TPC), total flavonoid content (TFC), TAC, and FRAP based on the protocol outlined in a previous study (16). TPC and TFC were measured using a microplate reader (BMG LABTECH, Ortenberg, Germany) at 700 nm and 415 nm, respectively, with gallic acid and quercetin as standards. TAC and FRAP were measured using Trolox as the standard (570 nm), and FRAP was examined using Fe (II) as the standard (560 nm).

### Cell culture and viability analysis

Mouse 3T3-L1 cells were purchased from ATCC, Manassas, VA, USA. Post-confluent cells were cultured in DMEM containing 10% calf serum and 1% penicillin/streptomycin. To examine viability, 1 × 10^4^ cells/well were incubated with various concentrations (0, 100, 250, 500, and 1,000 μg/mL) of kimchi ingredients for 24 h. Cells were reacted with CCK-8 solution for 2 h and measured at 450 nm.

To analyze the TG content, ORO staining intensity, and mRNA and protein expression, cells were differentiated in DMEM supplemented with 10% fetal bovine serum (FBS), 0.5 mM IBMX, 1 μM dexamethasone, 5 μg/mL insulin, and 500 μg/mL of kimchi ingredients for 2 days. Subsequently, they were maintained in DMEM with 10% FBS and 5 μg/mL insulin for 8 days.

In this study, the group of undifferentiated 3T3-L1 cells treated with DMSO alone (without differentiation or sample treatment) was defined as the negative control. The group of differentiated 3T3-L1 cells treated with DMSO (as the vehicle for the test samples) was defined as the experimental control. Each experimental group consisted of differentiated cells treated with freeze-dried kimchi sub-ingredients dissolved in DMSO.

### TG content analysis

The cells were treated with a H_2_O/chloroform/methanol mixture (3:4:8, v/v/v) at room temperature (15–25°C) for 1 h. The TG content of the bottom layer, which was dried overnight after centrifugation (800 × *g*, 10 min), was determined using a TG kit.

### ORO staining analysis

The cells were fixed and stained. The images of the stained cells were captured using a light microscope (10×, Olympus, Tokyo, Japan). ORO intensity was measured (510 nm) after extraction with isopropanol.

### mRNA expression analysis

The extracted total RNA was synthesized into cDNA, and a quantitative real-time polymerase chain reaction was conducted with the primers listed in Table 1 under the following conditions: activation for 10 min at 94°C, denaturation at 45 cycles for 15 s at 94°C, and annealing and extension for 1 min at 60°C. Data were standardized to those of glyceraldehyde-3-phosphate dehydrogenase (*GAPDH*).

**Table 1:** Primer sequences for quantitative real-time polymerase chain reaction

Gene	Forward primer (5′-3′)	Reverse primer (5′-3′)
*GAPDH*	GTATGACTCCACTCACGGCAAA	GGTGTGGCTCCTGGAAGATG
*aP2*	CATGGCCAAGCCCAACAT	CGCCCAGTTTGAAGGAAATC
*C/EBPα*	AGGTGCTGGAGTTGACCAGT	CAGCCTAGAGATCCAGCGAC
*FAS*	TCTGAGCAGGTGCAGGAGGA	GTTGTTCCTCCAGTTCCGATTTGTA
*LXRα*	CTCAATGCCTGATGTTTCTCCT	TCCAACCCTATCCCTAAAGCAA
*PPARγ*	TGGAATTAGATGACAGCGACTTGG	CTGGAGCAGCTTGGCAAACA
*SREBP-1c*	AGAGGGTGAGCCTGACAA	CCTCTGCAATTTCCAGAT

### Protein expression analysis

The extracted protein was collected through centrifugation (10,000 × *g* for 10 min at 4°C). Proteins (30 μg) were electrophoresed with 10% sodium dodecyl sulfate-gel and transferred onto the membranes (Bio-Rad, Hercules, CA, USA). Then, the membranes were reacted with antibodies (1:1,000 dilution). Protein expression was visualized using an ECL solution, and protein density was calculated using the Image J version 1.53 program (NIH, USA).

### Statistical analysis

Data are denoted as mean and standard deviation obtained in triplicate experiments. Data were processed using one-way analysis of variance (ANOVA), and significant differences were analyzed using Dunnett’s multiple comparison test (*P* < 0.05) in GraphPad Prism 9 (San Diego, CA, USA).

## Results

### Yields of kimchi ingredients according to the drying method

**Fig. 1:**
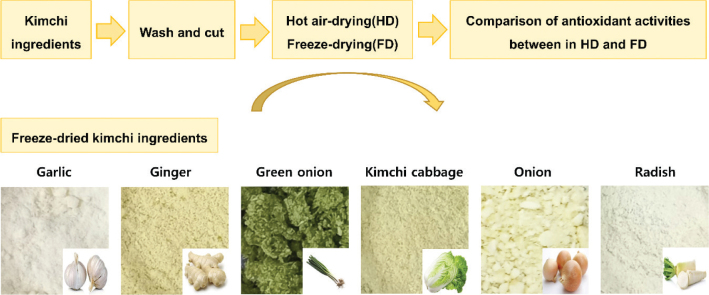
The process of preparing kimchi ingredients and the images of the freeze-dried kimchi ingredients.

In the screening test, the yield and the antioxidant effect of freeze-dried kimchi ingredients were higher than those of hot-air-dried kimchi ingredients (Table 2). Particularly, the yield of freeze-dried garlic was 318.07 g/kg, which was not only higher than the yield of hot-air-drying but also more than 5-fold higher than that of freeze-dried kimchi cabbage. Figure 1 shows the process of preparing kimchi ingredients and the image of the final selected freeze-dried kimchi ingredients. Among the kimchi ingredients, garlic had a high yield due to its low moisture content but demonstrated slight stickiness due to its high sugar content. Similar to garlic, onion also showed stickiness, appearing as a fine and slightly clumpy powder. These results suggest that freeze-drying could produce higher yields than the hot-air drying method.

**Table 2:** Yield and antioxidant activities of hot-air-dried (HD) and freeze-dried (FD) kimchi ingredients

	Garlic		Ginger		Green onion		Kimchi cabbage		Onion		Radish	
	HD	FD	HD	FD	HD	FD	HD	FD	HD	FD	HD	FD
*Yield (g/kg)*	310.64 ± 9.41^a^	318.07 ± 10.51^a^	94.79 ± 4.68^b^	97.59 ± 3.96^b^	80.41 ± 5.10^c^	93.03 ± 3.71^b^	55.57 ± 2.11^d^	59.67 ± 1.56^e^	77.16 ± 3.05^c^	85.72 ± 2.04^c^	76.22 ± 0.59^c^	77.54 ± 1.87^d^
*TPC (mg GAE/g)*	1.58 ± 1.28^f^	0.99 ± 0.10^f^	18.77 ± 2.90^a^	17.65 ± 0.59^a^	5.12 ± 1.46^d^	5.33 ± 0.24^d^	5.13 ± 0.6^c^	6.57 ± 0.11^c^	8.91 ± 1.45^b^	12.18 ± 0.09^b^	5.25 ± 2.28^e^	3.69 ± 0.10^e^
*TFC (mg QE/g)*	0.04 ± 0.05^e^	0.04 ± 0.10^e^	4.29 ± 0.39^a^	7.88 ± 0.33^a^	6.35 ± 0.38^c^	3.31 ± 0.05^c^	0.90 ± 0.10^d^	0.84 ± 0.25^d^	2.62 ± 0.10^b^	4.89 ± 0.30^b^	0.23 ± 0.09^de^	0.27 ± 0.09^de^
*TAC (nM)*	0.90 ± 0.00^f^	0.81 ± 0.00^f^	20.51 ± 0.71^a^	23.51 ± 0.53^a^	9.97 ± 0.06^d^	9.67 ± 0.12^d^	10.92 ± 0.14^c^	10.79 ± 0.08^c^	14.97 ± 0.06^b^	19.50 ± 0.26^b^	7.28 ± 0.00^e^	7.77 ± 0.04^e^
*FRAP (µM)*	142.87 ± 22.03^e^	189.53 ± 12.06^e^	2556.20 ± 53.81^a^	2830.87 ± 29.14^a^	769.53 ± 55.18^c^	856.20 ± 10.39^c^	782.87 ± 105.25^c^	766.87±68.89^c^	826.20±12.00^b^	1220.87±14.74^b^	210.87±16.17^d^	286.20±29.60^d^

Results are expressed as the mean ± SD.

Different lowercase letters indicate significant differences among groups with *P* < 0.05.

### Antioxidant activities of the kimchi ingredients according to the drying method

The antioxidant activities of the hot-air-dried and freeze-dried kimchi ingredients were compared by measuring TPC, TFC, TAC, and FRAP (Table 2). Most freeze-dried kimchi ingredients showed higher antioxidant activities than the hot-air-dried kimchi ingredients. Among kimchi ingredients, ginger and onion showed high activity, green onion and kimchi cabbage showed medium activity, and garlic and radish showed low activity in all antioxidant assays. Particularly, the TAC value of freeze-dried ginger was remarkably high, approximately 30-fold higher than that of freeze-dried garlic. Although the low antioxidant activities of garlic and radish were unexpected, the overall high antioxidant activity of kimchi ingredients suggests their potential lipid-lowering effects. Based on the yield and antioxidant activities results, we investigated the anti-obesity effect of freeze-dried kimchi ingredients at the cellular level.

### Effect of freeze-dried kimchi ingredients on the viability of 3T3-L1 preadipocytes

**Fig. 2:**
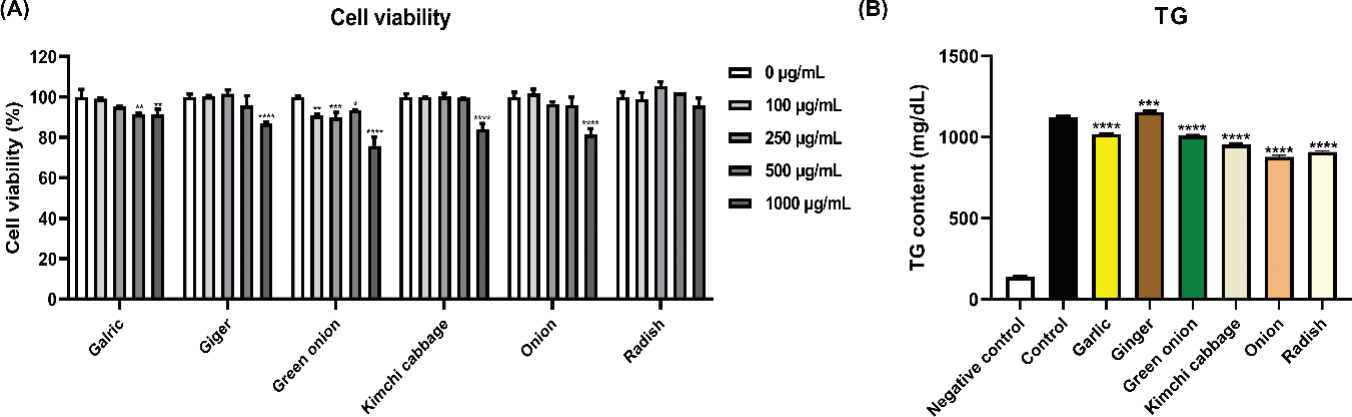
Effect of kimchi ingredients on (A) cell viability in 3T3-L1 preadipocytes and (B) triglyceride (TG) content in adipocytes. The results are expressed as mean ± SD. Negative control, undifferentiated 3T3-L1 cells treated with DMSO; Control, differentiated 3T3-L1 cells treated with DMSO. *P < 0.05, **P < 0.01, ***P < 0.001, and ****P < 0.0001 versus 0 μg/mL. ***P < 0.001 and ****P < 0.0001 versus Control.

The effects of freeze-dried kimchi ingredients on cell viability are displayed in Fig. 2A. Cell viability was over 90% when incubated with all ingredients at a concentration of 500 μg/mL, except green onion. At a concentration of 1,000 μg/mL, freeze-dried green onion exhibited approximately 75% cell viability, indicating cytotoxicity. In contrast, freeze-dried radish maintained cell viability over 95% at concentrations up to 1,000 μg/mL, indicating almost no cytotoxicity. These results indicate that the use of freeze-dried kimchi ingredients up to a concentration of 500 μg/mL is safe without cytotoxic effects on 3T3-L1 cells. Based on cell viability results, a concentration of 500 μg/mL of freeze-dried kimchi ingredients was selected for subsequent experiments.

### Effects of freeze-dried kimchi ingredients on the TG content in 3T3-L1 adipocytes

Figure 2B shows that all freeze-dried kimchi ingredients significantly decreased the TG content (*P* < 0.05). The TG content, which increased to 1124.03 mg/dL upon cell differentiation, was remarkably decreased to 877.92 mg/dL upon treatment with freeze-dried onion. Unexpectedly, the freeze-dried ginger exhibited TG content similar to that of the control (1124.03 mg/dL vs. 1154.65 mg/dL). The order of TG reduction effects was as follows: freeze-dried onion, radish, kimchi cabbage, green onion, and garlic. The TG reduction effect of freeze-dried ginger could not be confirmed. Considering the screening test results, freeze-dried kimchi cabbage and onion (500 μg/mL) were selected for the following experiments.

### Effects of freeze-dried kimchi cabbage and onion on lipid accumulation in 3T3-L1 adipocytes

**Fig. 3:**
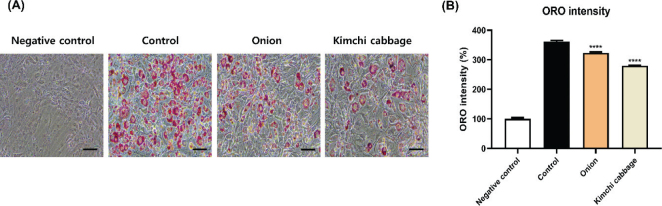
Effect of kimchi cabbage and onion (500 μg/mL) on lipid accumulation in 3T3-L1 adipocytes. (A) Oil red O staining images and (B) staining intensity. The results are expressed as mean ± SD. Negative control, undifferentiated 3T3-L1 cells treated with DMSO; Control, differentiated 3T3-L1 cells treated with DMSO. ****P < 0.0001 versus Control.

The effect of freeze-dried kimchi cabbage and onion on lipid accumulation was evaluated using ORO staining. Figure 3A and B show that freeze-dried kimchi cabbage and onion (500 μg/mL) significantly decreased lipid accumulation by 10 and 23%, respectively (*P* < 0.05). Freeze-dried kimchi cabbage inhibited lipid accumulation more efficiently than freeze-dried onion. This effect was assumed to be attributed to bioactive compounds and nutrients in freeze-dried kimchi cabbage and onion. The inhibitory effect of kimchi cabbage and onion on lipid accumulation indicates a lipid-lowering effect.

### Effect of freeze-dried kimchi cabbage and onion on obesity-associated mRNA expression in 3T3-L1 adipocytes

**Fig. 4:**
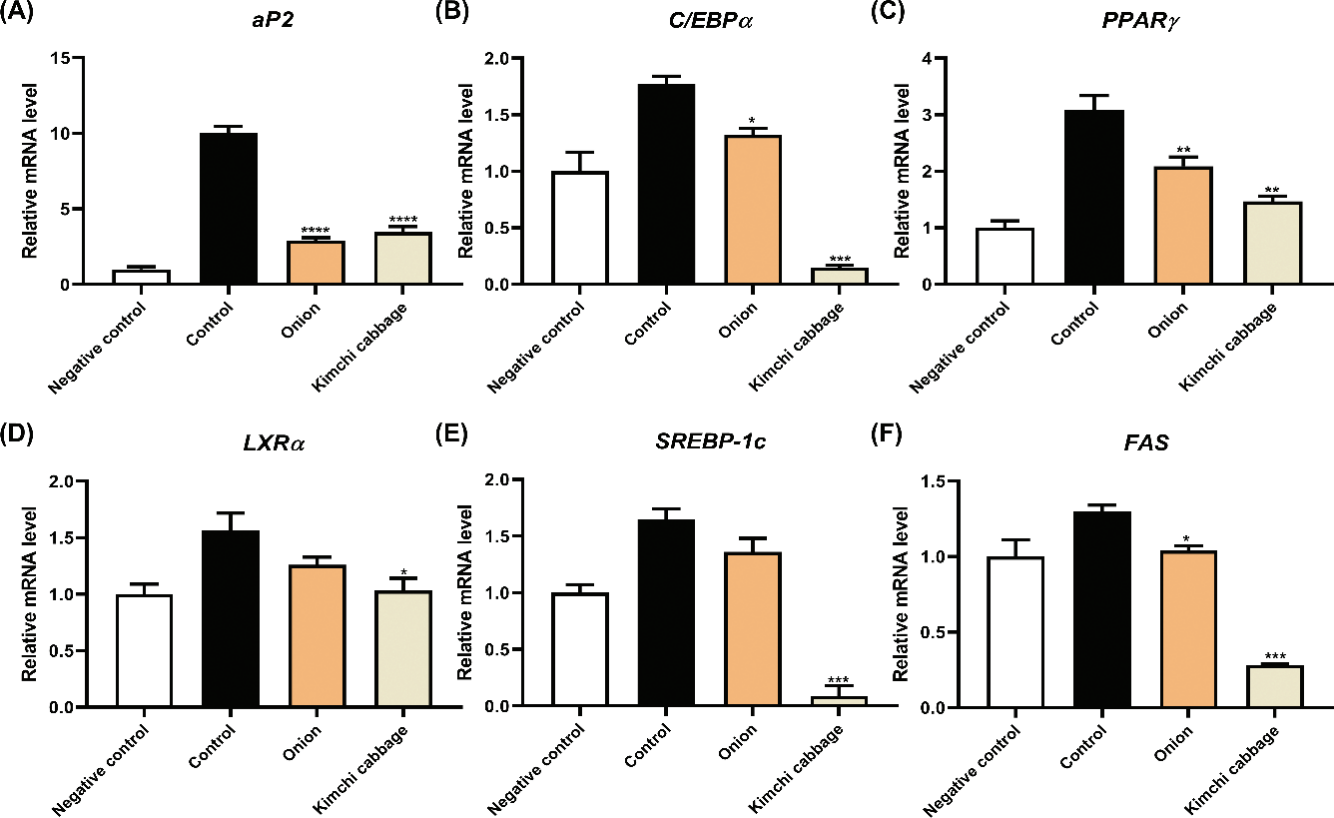
Effect of kimchi cabbage and onion (500 μg/mL) on obesity-associated mRNA expression in differentiated 3T3-L1 adipocytes. (A) *aP2*, (B) *C/EBP*α, (C) *PPAR*γ, (D) *LXR*α, (E) *SREBP-1c*, and (F) *FAS*. The results are expressed as mean ± SD. Negative control, undifferentiated 3T3-L1 cells treated with DMSO; Control, differentiated 3T3-L1 cells treated with DMSO. *P < 0.05, **P < 0.01, and ***P < 0.001 versus Control.

Figure 4 shows the effects of freeze-dried kimchi cabbage and onion on adipogenic and lipogenic mRNA expression (Fig. 4). Among adipogenic biomarkers, freeze-dried kimchi cabbage and onion particularly decreased the mRNA expression of adipocyte fatty acid-binding protein (*aP2*), CCAAT/enhancer-binding protein α (*C/EBP*α), and *PPAR*γ compared to that observed in the control (Fig. 4A–C). Among lipogenic biomarkers, freeze-dried kimchi cabbage significantly decreased the mRNA expression of *SREBP-1c* and *FAS* compared to that observed in the control (Fig. 4D–E, *P* < 0.001). With respect to obesity-associated biomarkers, freeze-dried kimchi cabbage exhibited a more potent inhibitory effect than freeze-dried onion. Together, freeze-dried kimchi cabbage and onion regulated adipogenesis and lipogenesis via the C/EBPα and PPARγ pathways, highlighting their potential to inhibit lipid accumulation and adipocyte differentiation.

### Effect of freeze-dried kimchi cabbage and onion on obesity-associated protein expression in 3T3-L1 adipocyte

**Fig. 5:**
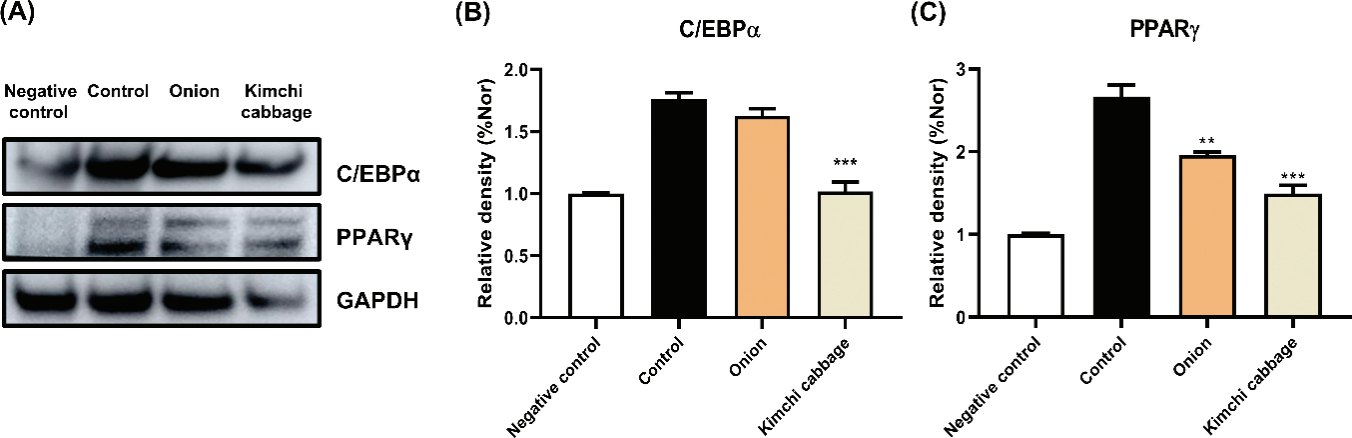
Effect of kimchi cabbage and onion (500 μg/mL) on obesity-associated protein expression in differentiated 3T3-L1 adipocytes. (A) Western blot image, (B) C/EBPα expression levels, and (C) PPARγ expression levels. The results are expressed as mean ± SD. Negative control, undifferentiated 3T3-L1 cells treated with DMSO; Control, differentiated 3T3-L1 cells treated with DMSO. **P < 0.01, ***P < 0.001, and ****P < 0.0001 versus Control.

We investigated the protein expression of C/EBPα and PPARγ to validate the effects of freeze-dried kimchi cabbage and onion on lipid metabolism. As shown in Fig. 5A–C, freeze-dried kimchi cabbage and onion significantly decreased C/EBPα and PPARγ protein expression, consistent with mRNA expression results (*P* < 0.05). Based on mRNA and protein expression, we concluded that the freeze-dried kimchi cabbage and onion modulated obesity via regulation in the C/EBPα and PPARγ pathways.

## Discussion

As mentioned earlier, the health benefits of extracts or fractions derived from kimchi ingredients have been continuously studied. However, since the raw materials themselves may exhibit cytotoxicity or lack functionality when directly applied to cells, this study focuses on the potential of freeze-dried kimchi materials rather than their unprocessed, raw form. Freeze-drying is expected to preserve the physiologically active compounds of each ingredient while maintaining their functional properties. Therefore, this study aimed to confirm the anti-obesity effects of freeze-dried kimchi ingredients rather than extracts or fractions of kimchi ingredients as previously studied. Hence, the antioxidant activities of six kimchi ingredients were compared after hot-air-drying and freeze-drying, and the anti-obesity effect of kimchi ingredients with high antioxidant activity was confirmed using 3T3-L1 adipocytes.

Most kimchi ingredients exhibit high moisture content. The yield varies depending on the difference in moisture content. The moisture content of the six kimchi ingredients ranges from 80 to 95%, except for garlic. The moisture content of kimchi cabbage is the highest at approximately 95%, and that of garlic is the lowest at approximately 63% (17). After drying, the powder form may appear fine or lumpy depending on the sugar content and moisture adsorption capacity. For instance, freeze-dried samples show moisture sorption characteristics in the air (18). In this study, a slightly lumpy form was observed with green onion and onion due to high sugar content and high moisture sorption properties. Along with yields, Fig. 1A shows images of six freeze-dried kimchi ingredients.

According to previous studies, antioxidant activities vary depending on the type of ingredients and the processing methods (19–21). For instance, the functional effects differ depending on the form of the garlic, and garlic powder shows low antioxidant and antiproliferative activities (19). In the present study, unexpectedly low antioxidant activities of hot-air-dried and freeze-dried garlic are likely attributable to the processing method. In another study, Leelarungrayub et al. reported that freeze-dried garlic hexane extract showed high antioxidant activity (20). When subjected to different drying methods, ginger showed high antioxidant activities, with sun-dried showing the highest, followed by oven-dried and freeze-dried ginger (21). Although the low antioxidant activities of garlic and radish were unexpected, the overall high antioxidant activity of kimchi ingredients suggests their potential to regulate lipid metabolism and adipogenesis.

Previous studies on kimchi ingredients have primarily focused on bioactive compounds, such as alliin, gingerol, and quercetin. For instance, engelitin, quercetin, and caffeic acid in *Smilax china* L. were used at 40 μg/mL owing to the lack of cytotoxicity at this concentration (22). We investigated the cell viability of freeze-dried kimchi ingredients in this study. A total of 6 freeze-dried kimchi ingredients showed over 90% cell viability up to 500 μg/mL, demonstrating low cytotoxicity. Consistent with these findings, freeze-dried kimchi exhibited more than 80% cell viability up to a 1,000 μg/mL concentration in a previous study (1). Interestingly, freeze-dried kimchi was safe without toxic effects up to 2,500 μg/mL in RAW264.7 cells (23). Based on cell viability results, the anti-obesity effect of freeze-dried kimchi could be investigated with 500 μg/mL, as it exhibits low cytotoxicity at this concentration.

To investigate the TG-reducing effect, the TG content in 3T3-L1 adipocytes was measured after treatment with six freeze-dried kimchi ingredients. Inconsistent with antioxidant activities, the TG reduction effect of freeze-dried ginger was rather low. Except for ginger, five freeze-dried kimchi ingredients significantly decreased TG content compared to the control (Fig. 2B, *P* < 0.05). Similar to our results, garlic and onion dose-dependently decreased TG content in previous studies (24, 25). Lee et al. reported that raw garlic extract showed the highest TG reduction effect among picked-, vinegar picked-, and raw garlic butanol extracts (24). Onion peel ethanol extract also significantly decreased the TG content (25). Based on the results of cell viability and TG content, 500 μg/mL of freeze-dried kimchi cabbage and onion were selected for use in the next experiment to examine the anti-obesity effect.

The results showed the inhibitory effect of freeze-dried kimchi cabbage and onion on lipid accumulation. This effect was hypothesized to be attributed to bioactive compounds and nutrients in freeze-dried kimchi cabbage and onion. Quercetin, the active compound in onion, significantly decreased lipid accumulation in 3T3-L1 adipocytes (22). Similarly, sulforaphane, the bioactive compound of kimchi cabbage, efficiently suppressed lipid accumulation in cells and rats (26, 27). Moreover, freeze-dried kimchi powder prepared with kimchi ingredients significantly decreased lipid accumulation in 3T3-L1 adipocytes and obese mice (1, 4). The lipid accumulation inhibitory effect of freeze-dried kimchi cabbage and onion suggests their potential to regulate lipid metabolism and adipogenesis.

Freeze-dried kimchi cabbage and onion significantly reduced adipogenic and lipogenic mRNA expression (*P* < 0.05). This reduction was more pronounced with kimchi cabbage. In some studies, onion peel extract significantly downregulated adipogenic mRNA expression because of the abundance of quercetin (25, 28). Consistent with the adipogenic biomarkers, freeze-dried kimchi cabbage and onion markedly decreased the mRNA expression of *LXR*α, sterol regulatory element-binding protein (*SREBP-1c*), and fatty acid synthase (*FAS*) (Fig. 4D and 4). According to Lu et al., bioactive compounds extracted from onions modulate lipogenesis (10). Freeze-dried kimchi also showed significant adipogenic and lipogenic mRNA expression (1). Freeze-dried kimchi cabbage exhibited a stronger inhibitory effect than onion.

C/EBPα and PPARγ play critical roles in obesity via modulation of the expression of the adipogenic biomarkers (29). Therefore, studies on C/EBPα and PPARγ protein expression have been extensively conducted (30, 31). For instance, sesamin and sesamolin, major sesame lignans, controlled obesity by downregulating C/EBPα and PPARγ expression (30). To confirm the anti-obesity effect of freeze-dried kimchi cabbage and onion, the obesity-associated protein expression was investigated. Freeze-dried kimchi cabbage and onion significantly decreased C/EBPα and PPARγ protein expression, consistent with previous kimchi study results (1). These results suggest that freeze-dried kimchi cabbage and onion exert anti-obesity effects by regulating the C/EBPα and PPARγ pathways.

Taken together, the findings show that freeze-dried kimchi cabbage and onion modulated obesity by regulating adipogenesis and lipogenesis via the C/EBPα and PPARγ pathways.

Despite our promising results, this study had some limitations. One limitation of this study is the absence of a conventional positive control group, which may affect the interpretation of the results. We explored the antioxidant and lipid-lowering effects of freeze-dried kimchi ingredients, especially kimchi cabbage and onion *in vitro*; further studies are necessary to validate these effects *in vivo*. Future mechanistic studies are required to determine the individual components responsible for these effects. Further research on the specific mechanism of action is also warranted. Based on these results, follow-up research will be conducted.

## Conclusions

In this study, the antioxidant and lipid-lowering effects of six kimchi ingredients were identified. Freeze-dried kimchi cabbage and onion demonstrated the most potent antioxidant effects, reduced TG content, and no cytotoxicity during the screening test. Furthermore, freeze-dried kimchi cabbage and onion regulated lipid metabolism by inhibiting lipid accumulation and modulating obesity-associated mRNA/protein expression related to the C/EBPα and PPARγ pathways. Based on these results, we validated that the health benefits of kimchi, including antioxidant and lipid-lowering effects, are attributed to kimchi ingredients. In addition, the antioxidant and lipid-lowering effects of functional kimchi ingredients could pave the way for its application in various fields such as medicine and pharmacy.

## Data Availability

Not applicable.
